# Exploring the impact of selection bias in observational studies of COVID-19: a simulation study

**DOI:** 10.1093/ije/dyac221

**Published:** 2022-12-06

**Authors:** Louise A C Millard, Alba Fernández-Sanlés, Alice R Carter, Rachael A Hughes, Kate Tilling, Tim P Morris, Daniel Major-Smith, Gareth J Griffith, Gemma L Clayton, Emily Kawabata, George Davey Smith, Deborah A Lawlor, Maria Carolina Borges

**Affiliations:** MRC Integrative Epidemiology Unit, University of Bristol, Bristol, UK; Population Health Sciences, Bristol Medical School, University of Bristol, Bristol, UK; MRC Integrative Epidemiology Unit, University of Bristol, Bristol, UK; Population Health Sciences, Bristol Medical School, University of Bristol, Bristol, UK; MRC Integrative Epidemiology Unit, University of Bristol, Bristol, UK; Population Health Sciences, Bristol Medical School, University of Bristol, Bristol, UK; MRC Integrative Epidemiology Unit, University of Bristol, Bristol, UK; Population Health Sciences, Bristol Medical School, University of Bristol, Bristol, UK; MRC Integrative Epidemiology Unit, University of Bristol, Bristol, UK; Population Health Sciences, Bristol Medical School, University of Bristol, Bristol, UK; NIHR Biomedical Research Centre, Bristol, UK; MRC Clinical Trials Unit, University College London, London, UK; MRC Integrative Epidemiology Unit, University of Bristol, Bristol, UK; Population Health Sciences, Bristol Medical School, University of Bristol, Bristol, UK; MRC Integrative Epidemiology Unit, University of Bristol, Bristol, UK; Population Health Sciences, Bristol Medical School, University of Bristol, Bristol, UK; MRC Integrative Epidemiology Unit, University of Bristol, Bristol, UK; Population Health Sciences, Bristol Medical School, University of Bristol, Bristol, UK; MRC Integrative Epidemiology Unit, University of Bristol, Bristol, UK; Population Health Sciences, Bristol Medical School, University of Bristol, Bristol, UK; MRC Integrative Epidemiology Unit, University of Bristol, Bristol, UK; Population Health Sciences, Bristol Medical School, University of Bristol, Bristol, UK; NIHR Biomedical Research Centre, Bristol, UK; MRC Integrative Epidemiology Unit, University of Bristol, Bristol, UK; Population Health Sciences, Bristol Medical School, University of Bristol, Bristol, UK; NIHR Biomedical Research Centre, Bristol, UK; MRC Integrative Epidemiology Unit, University of Bristol, Bristol, UK; Population Health Sciences, Bristol Medical School, University of Bristol, Bristol, UK

**Keywords:** Selection bias, misclassification bias, SARS-CoV-2 infection, COVID-19, UK Biobank, ALSPAC

## Abstract

**Background:**

Non-random selection of analytic subsamples could introduce selection bias in observational studies. We explored the potential presence and impact of selection in studies of SARS-CoV-2 infection and COVID-19 prognosis.

**Methods:**

We tested the association of a broad range of characteristics with selection into COVID-19 analytic subsamples in the Avon Longitudinal Study of Parents and Children (ALSPAC) and UK Biobank (UKB). We then conducted empirical analyses and simulations to explore the potential presence, direction and magnitude of bias due to this selection (relative to our defined UK-based adult target populations) when estimating the association of body mass index (BMI) with SARS-CoV-2 infection and death-with-COVID-19.

**Results:**

In both cohorts, a broad range of characteristics was related to selection, sometimes in opposite directions (e.g. more-educated people were more likely to have data on SARS-CoV-2 infection in ALSPAC, but less likely in UKB). Higher BMI was associated with higher odds of SARS-CoV-2 infection and death-with-COVID-19. We found non-negligible bias in many simulated scenarios.

**Conclusions:**

Analyses using COVID-19 self-reported or national registry data may be biased due to selection. The magnitude and direction of this bias depend on the outcome definition, the true effect of the risk factor and the assumed selection mechanism; these are likely to differ between studies with different target populations. Bias due to sample selection is a key concern in COVID-19 research based on national registry data, especially as countries end free mass testing. The framework we have used can be applied by other researchers assessing the extent to which their results may be biased for their research question of interest.

Key Messages

- Observational studies assessing the association of risk factors with health outcomes are often restricted to a much smaller subsample of the original cohort, which could result in a non-random sample of the target population and therefore spurious associations.
- Our results demonstrate that, in studies of SARS-CoV-2 infection and COVID-19 prognosis, selection into analytical subsamples can induce non-negligible bias.
- Researchers should conduct sensitivity analyses and simulations to explore the robustness of their results to different selection mechanisms.
- We provide a framework that is applicable beyond COVID-19 research.

## Introduction

Analyses using large-scale observational studies are often conducted on non-random subsamples of the target population—the group that inferences are to be made about^1^—e.g. due to non-random study recruitment or loss to follow-up.^2^^,^^3^ Selection bias can occur when the study sample does not represent the target population and therefore affects the external validity of the causal effect (i.e. the true causal effect in the study sample is different from the true causal effect in the target population).^1^ Additionally, when both the exposure and outcome (or a cause of these) influence the probability of being selected (from the study sample) into the analytical sample, selection-induced collider bias can occur.^2^^,^^3^ This can induce an association between the exposure and outcome when none exists in the whole sample, or attenuate, inflate or reverse the estimated effect of the exposure on the outcome in the selected subsample.^4^^,^^5^ Confounding can also be present in an observational study and can also attenuate, inflate or reverse the estimated effect of the exposure on the outcome in the analytical sample. Both confounding and selection can be present (as is likely in our examples), affecting the internal validity of the causal effect (i.e. the causal effect estimated in the analytical sample is different from the true causal effect in the study sample).^1^

Selection bias may be a particular cause for concern in research investigating determinants of SARS-CoV-2 infection or COVID-19 prognosis. These studies frequently rely on samples of individuals who volunteered to participate in COVID-19 substudies, were tested for SARS-CoV-2 infection or were admitted to a hospital. Furthermore, misclassification of cases and non-cases of SARS-CoV-2 infection due to selection (which we refer to as selection-induced misclassification bias) is another key potential source of bias. This may occur in studies using ‘population-based comparison groups’ in which all individuals that are not known cases (including those with missing data on infection status) are included in the comparison group.^6–9^ As an example, the COVID-19 Host Genetics Initiative, a large-scale collaboration focused on understanding genetic determinants of SARS-CoV-2 infection or COVID-19 prognosis, uses ‘population-based comparison groups’.^6^ However, little attention has been given to potential implications of these definitions.

We aimed to explore selection-induced collider and misclassification bias when estimating the association of risk factors for SARS-CoV-2 infection and the prognostic factors of COVID-19, using data from two UK cohort studies, and both empirical analyses and simulations.

## Methods

### Prospective cohort studies

#### Avon Longitudinal Study of Parents and Children

The multigenerational Avon Longitudinal Study of Parents and Children (ALSPAC) birth cohort initially recruited 14 541 pregnancies (∼75% response of eligible women), who gave birth to 14 062 children in the former county of Avon in the Southwest of England in 1991–1992.^10^^,^^11^ Mothers and children have been followed up with regular assessments. When the oldest children were 7 years old, a further 913 eligible children (also born in the Southwest of England in 1991–1992) were enrolled;^12^ 14 849 of the index children (aged 29–31 years at the most recent follow-up) who were alive at 1 year old and had not withdrawn from ALSPAC were eligible for analyses. Ethical approval was obtained from the ALSPAC Ethics and Law Committee and the local research ethics committees under project B3543.

From April 2020, participants were sent four questionnaires to collect self-reported information relevant to the COVID-19 pandemic and its consequences, including COVID-19 status, behavioural, lifestyle and health-related factors.^13–17^ Our analyses focus on the first COVID-19 questionnaire (Q1), sent between 9 April and 14 May 2020.^13^ SARS-CoV-2 infection was ascertained by asking participants ‘Do you think that you have, or have had, COVID-19?’, where participants could respond (a) yes, confirmed by a positive test; (b) yes, doctor’s suspicion; (c) yes, own suspicion; or (d) do not think they had COVID-19. Of the 14 849 eligible participants, 2966 responded to that question (Supplementary Figure 1a, available as Supplementary data at *IJE* online).

We define our target population as young adults (aged 20–40 years in March 2020) resident in the UK during the COVID-19 pandemic. SARS-CoV-2 infection [‘SARS-CoV-2(+)’] was defined as participants who responded either ‘a’, ‘b’ or ‘c’ to the above question.^13^ Two control groups were defined: (i) participants who responded ‘d’ [‘SARS-CoV-2(−)’] and (ii) those who responded ‘d’ or did not respond to that question or did not receive or return the questionnaire (‘everyone else’). COVID-19 prognosis could not be studied in ALSPAC as this was not assessed in Q1 and death registry data were not available.^13^

#### UK Biobank

UK Biobank (UKB) recruited 503 317 UK adults (aged 37–73 years) from 22 centres across England, Wales and Scotland from 2006 to 2010 (5.5% response rate).^18^ Participants attended baseline assessment centres and follow-up data were obtained from (limited) clinics and questionnaires, and linkage to national registries.^18^^,^^19^ We used data from baseline with linked hospital episode statistics, mortality statistics and Public Health England test results for active SARS-CoV-2 infection. UKB provided ethical approval for UKB project 16729; 421 037 participants who were residents in England at baseline and alive on 1 January 2020 were eligible for analyses (Supplementary Figure 1b, available as Supplementary data at *IJE* online).

We define our target population as middle-aged and elderly adults (aged 40–70 years in March 2020) resident in the UK during the COVID-19 pandemic. SARS-CoV-2 infection [‘SARS-CoV-2(+)’] was defined as either a positive polymerase chain reaction (PCR) test or COVID-19 recorded on a death certificate between 1 January and 18 May 2020. This cut-off date was chosen as it was the day mass testing became available.^20^ COVID-19 deaths were defined using International Classification of Diseases 10th revision (ICD-10) codes U07.1 (laboratory-confirmed COVID-19) and ICD-10 code U07.2 (a clinical or epidemiological diagnosis of COVID-19).^21^ Two control groups were defined: (i) participants with a negative PCR test [‘SARS-CoV-2(−)’] and (ii) participants with a negative PCR test or no PCR test record (‘everyone else’).

COVID-19 prognosis was defined using COVID-19 deaths. COVID-19 could be either the primary or contributory cause of death (i.e. they could have died ‘from’ COVID-19 or ‘with’ COVID-19). We therefore refer to ‘death-with-COVID-19’ throughout. Control groups for death-with-COVID-19 were defined as: (i) SARS-CoV-2(+) participants who did not die with COVID-19 and (ii) SARS-CoV-2(+) participants who did not die with COVID-19, or SARS-CoV-2(−) or untested participants.

### Statistical analyses

#### Association of candidate predictors of selection and SARS-CoV-2 infection

In both cohorts we used univariable logistic regression to test whether a wide range of characteristics predicted being selected into the analytical subsample (i.e. having data on SARS-CoV-2 infection compared with having no data, referred to as ‘assessed’ vs ‘non-assessed’). These characteristics included socio-demographic (deprivation indices and education), behavioural (alcohol intake and smoking) and health-related [pre-existing conditions, body mass index (BMI) and blood pressure] factors (Supplementary Table 1, available as Supplementary data at *IJE* online). Individuals were included if they had complete data on the variable under analysis, so the sample in each analysis differs.

#### Association of BMI with SARS-CoV-2 infection and death-with-COVID-19

We used multivariable logistic regression comparing SARS-CoV-2(+) with the two control groups, adjusting for age, sex, smoking status, educational attainment and deprivation indices (Index of Multiple Deprivation in ALSPAC and Townsend deprivation index in UKB). Individuals with complete data on BMI, COVID-19 outcomes and covariates were included.

#### Simulation study exploring bias in empirical estimate

We performed two simulation studies and below use the aims, data‐generating mechanisms, estimands, methods and performance measures (ADEMP) approach to report these simulations.^22^

##### Simulation A: Examining the bias in estimating the association of BMI with assessed as positive for SARS-CoV-2 infection

Aim: Assess the bias that may occur when estimating the association of BMI with SARS-CoV-2 infection (conditional on confounders) when only a subsample of participants has a SARS-CoV-2 infection assessment.

**Figure 1:**
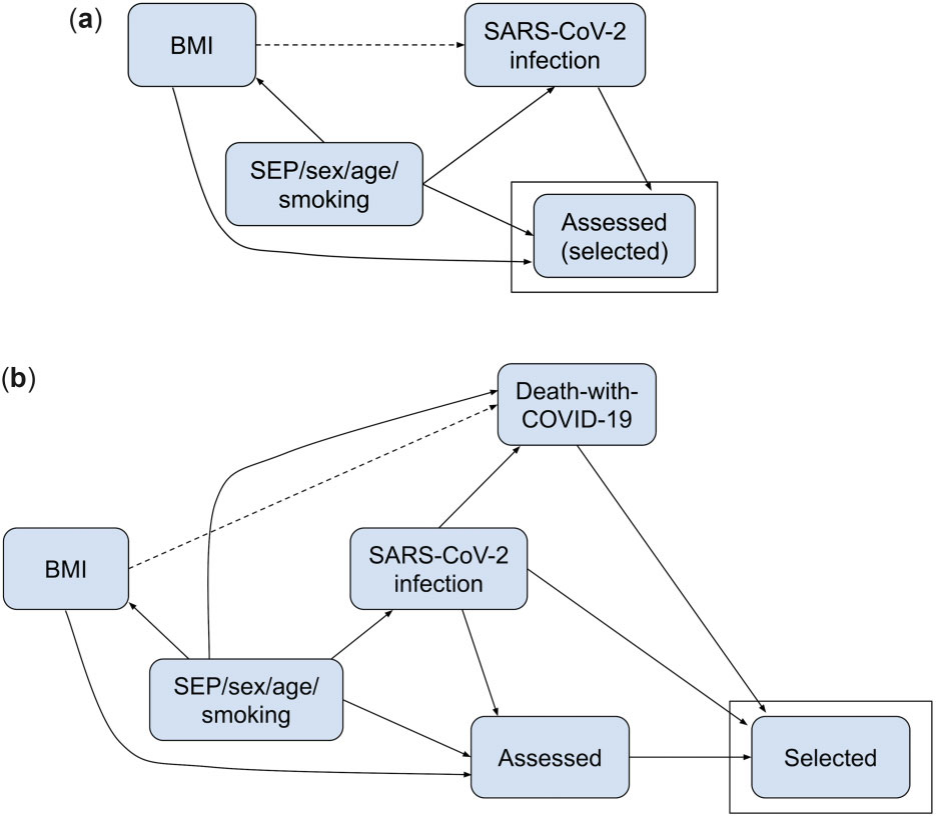
Directed acyclic graphs depicting assumed causal models for empirical and simulation scenarios. (a) SARS-CoV-2 infection. Dashed lines indicate the causal effect we are estimating. Simulations based on Avon Longitudinal Study of Parents and Children (ALSPAC) and UK Biobank (UKB) data. Participants were assessed (and hence selected) if they reported whether they have had a SARS-CoV-2 infection in ALSPAC or had a SARS-CoV-2 polymerase chain reaction (PCR) test result in UKB. (b) Death-with-COVID-19. Dashed lines indicate the causal effect we are estimating. Simulations based on UKB data only. Participants were selected if they were assessed [as in (a)] and tested positive or if they died with COVID-19. An arrow from Node A to Node B in a directed acyclic graph (DAG) indicates that A is a direct cause of B (i.e. A affects B not only through another node in the DAG). DAGs do not describe ‘how’ this effect occurs, i.e. the specific model describing this relationship, including whether nodes interact in their effects. For example, in DAG (b), infection is a direct cause of death-with-COVID-19 as a person can only die with COVID-19 if they are infected. Thus, infection interacts with all other direct effects of death-with-COVID-19. For example, smoking directly affects the risk of dying with COVID-19 only among those with a SARS-CoV-2 infection (i.e. the effect of smoking on death-with-COVID-19 depends on SARS-CoV-2 infection status). BMI, body mass index; SEP, socio-economic position.

Data-generating mechanism: The data-generating mechanism was based on the directed acyclic graph (DAG) shown in Figure 1a. We simulated data-set sample sizes of 14 849 (ALSPAC), and 421 037 (UKB) to reflect the empirical data. Parameters of the models used to generate the simulated data were based on estimated values from ALSPAC and UKB, and statistics from the published literature (Supplementary Sections 1 and 2, and Supplementary Tables 4 and 5, available as Supplementary data at *IJE* online). We repeated the simulations assuming (i) no effect of BMI on SARS-CoV-2 infection and (ii) a strong effect (OR=3) of BMI on SARS-CoV-2 infection. Selection in Simulation A was defined as participants who were assessed for SARS-CoV-2 infection. We induced selection bias by including an additive interaction effect on the log probability scale of BMI with SARS-CoV-2 infection on selection (Supplementary Sections 1, 2 and 4, available as Supplementary data at *IJE* online). We simulated three scenarios to induce different magnitudes of selection bias, with the following effect of BMI and SARS-CoV-2 infection on selection: (i) no interaction (main effects only on the log probability scale), (ii) ‘plausible’ interaction effect [log risk ratio (RR)=0.0527 in ALSPAC- and –0.162 in UKB-based scenarios] and (iii) ‘extreme’ interaction effect (log RR=0.135 in ALSPAC- and –0.245 in UKB-based scenarios). For each of these scenarios, the main effect and intercept were adjusted such that the total effect of BMI and SARS-CoV-2 infection on the selection remained constant (Supplementary Section 4, available as Supplementary data at *IJE* online).

Target estimand: The odds ratio (OR) of SARS-CoV-2 infection per SD increase in BMI, conditional on confounders (but not conditional on selection).

Methods: We evaluated two outcome definitions to estimate the association of BMI with SARS-CoV-2 infection using logistic regression:

- SARS-CoV-2(+) vs SARS-CoV-2(−);
- SARS-CoV-2(+) vs ‘everyone else’ [i.e. SARS-CoV-2(−) and non-assessed].

We used Wald-type confidence intervals on the log odds scale with the standard error (SE) taken from the inverse estimated information matrix.

Performance measure: We estimated the bias (and Monte Carlo SE; MCSE) of the estimated effect of BMI on SARS-CoV-2 infection compared with the true value (for each of the above methods). We estimated the confidence interval coverage (the proportion of repetitions where the confidence intervals included the true value). These performance measures were estimated across 1000 simulation repetitions (Supplementary Section 5, available as Supplementary data at *IJE* online).

##### Simulation B: Examining the bias in estimating the association of BMI with death-with-COVID-19

Aim: Assess bias due to selection in estimates of the association of BMI with death-with-COVID-19 (recalling that Simulation A considered infection).

Data-generating mechanism: The data-generating mechanism was based on the DAG shown in Figure 1b. The confounders, BMI, SARS-CoV-2 infection and being assessed (selection in Simulation A) were generated as described in Simulation A. Selection in Simulation B was defined as participants who were either assessed and were SARS-CoV-2(+) or those who died with COVID-19. As in Simulation A, model parameters used were either estimated in UKB or extracted from published literature (Supplementary Section 1 and Supplementary Tables 4 and 5, available as Supplementary data at *IJE* online). We repeated the simulation assuming: (i) no effect of BMI on death-with-COVID-19 and (ii) OR=3 effect of BMI on death-with-COVID-19. Further details are provided in Supplementary Sections 1–4 and Supplementary Tables 4 and 5 (available as Supplementary data at *IJE* online).

Target estimand: OR of death-with-COVID-19 per SD increase in BMI, conditional on confounders and having a SARS-CoV-2 infection (but not conditional on being assessed).

Methods: We evaluated two outcome definitions to estimate the association of BMI with death-with-COVID-19 using logistic regression:

- Died with COVID-19 vs SARS-CoV-2(+) who did not die with COVID-19;
- Died with COVID-19 vs ‘everyone else’.

Performance measure: We assessed bias, MCSE and coverage compared with the true value.

Analyses were performed in R version 3.5.1 or Stata version 15 and analysis code is available at https://github.com/MRCIEU/COVIDITY_selbias/. Git tag v0.1 corresponds to the version of the analyses presented here.

## Results

### Sample characteristics

#### ALSPAC

In total, 2966 out of 14 849 ALSPAC participants were assessed for COVID-19; 72% of them (2122) were females with a mean age of 27.6 years (SD = 0.54) (Supplementary Table 2A, available as Supplementary data at *IJE* online). With the exceptions of age and sex, all candidate predictors of selection had some missing data (range 11–74%; Supplementary Table 3, available as Supplementary data at *IJE* online).

#### UKB

In total, 4869 out of 421 037 participants in UKB were assessed for COVID-19; 51% of them (2496) were female with a mean age of 57.1 years (SD=8.9) (Supplementary Table 2B, available as Supplementary data at *IJE* online). One per cent of participants had a recorded SARS-CoV-2 test or death, where 30% of the tests were positive (Supplementary Table 2B, available as Supplementary data at *IJE* online). Few candidate predictors of selection had missing data (Supplementary Table 3, available as Supplementary data at *IJE* online).

### Association between candidate predictors of selection and SARS-CoV-2 infection

#### ALSPAC

**Figure 2:**
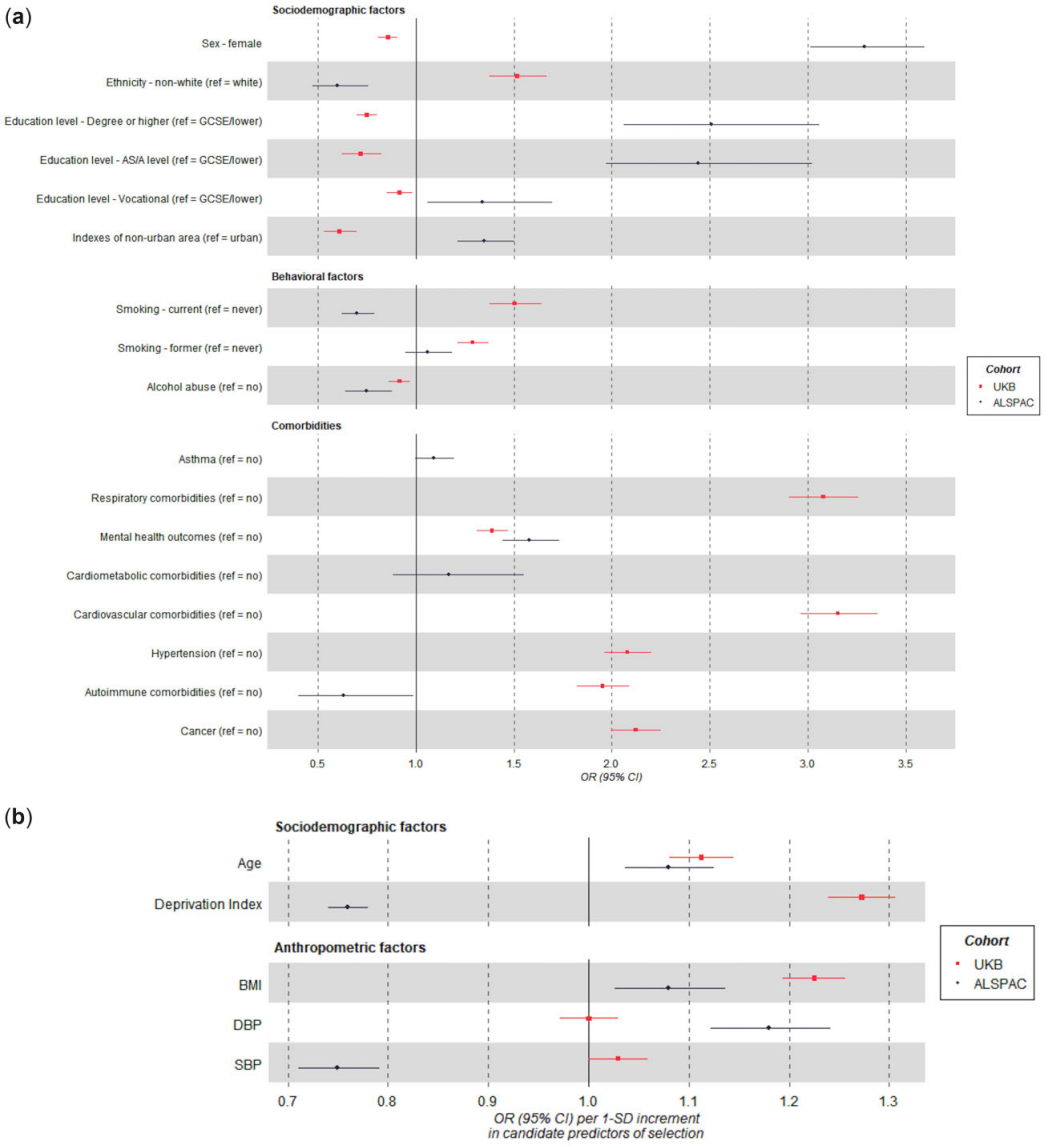
Forest plots of the association between the candidate predictors of selection and outcomes related to SARS-CoV-2 infection. ORs and their 95% CIs are shown for (a) categorical variables and (b) continuous variables. Estimates for continuous candidate predictors are per 1 SD for each predictor except for the Deprivation Index, which is given per 1 higher quantile. ALSPAC, Avon Longitudinal Study of Parents and Children; UKB, UK Biobank; OR, odds ratio; BMI, body mass index; SBP, systolic blood pressure; DBP, diastolic blood pressure; GCSE, General Certification of Secondary Education.

Most of the candidate predictors of selection were associated with being assessed (having self-reported data) for SARS-CoV-2 infection compared with not being assessed. Females, older participants, with higher education attainment, living in non-urban areas, suffering from adverse mental health outcomes and with higher BMI and diastolic blood pressure were more likely to be assessed (e.g. OR=3.29 for sex, 95% CI: 3.01, 3.59). Non-White participants, current smokers and those having a history of alcohol abuse, living in more deprived areas and suffering from some autoimmune co-morbidities were less likely to be assessed (e.g. OR=0.60 for ethnicity, 95% CI: 0.47, 0.75) (Figure 2).

#### UKB

Except for diastolic blood pressure, all variables were associated with being assessed (tested) for SARS-CoV-2 infection compared with not being assessed. Variables associated with a higher odds of being assessed included being older, reporting non-White ethnicity, being a former or current smoker, having higher BMI and pre-existing conditions (e.g. OR=3.15 for a previous cardiovascular diagnosis, 95% CI: 2.96, 3.35). Females and participants living in a rural area and having higher educational attainment were less likely to be assessed (e.g. OR=0.75 for leaving education with a degree or more compared with General Certificate of Secondary Education or less, 95% CI: 0.70, 0.80) (Figure 2).

### Association between BMI and SARS-CoV-2 infection and death-with-COVID-19

#### ALSPAC

**Figure 3:**
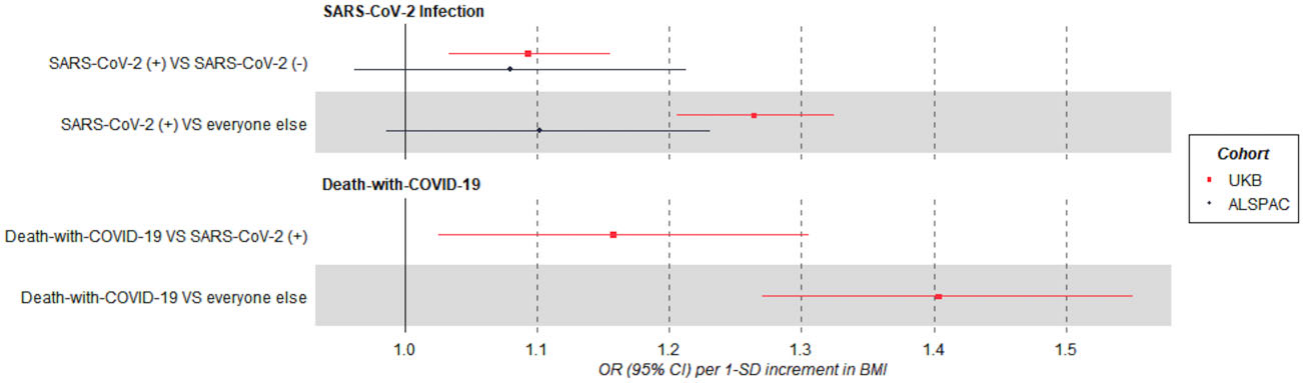
Forest plots of the association between BMI and COVID-19-related outcomes. In the ALSPAC cohort of young adults; SARS-CoV-2(+) vs SARS-CoV-2(−) *N*=1915, SARS-CoV-2(+) vs ‘everyone else’ *N*=2983. In UKB; SARS-CoV-2(+) vs SARS-CoV-2(−) *N*=4662, SARS-CoV-2(+) vs ‘everyone else’ *N*=409 487. Death-with-COVID-19 vs SARS-CoV-2(+) not resulting in death-with-COVID-19 *N*=1375, death-with-COVID-19 vs ‘everyone else’ *N*=409 487. Models were adjusted for age, sex, smoking, education and proxies of socio-economic position. ‘Everyone else’ control group includes those tested and SARS-CoV-2(−) and those not tested. BMI, body mass index; ALSPAC, Avon Longitudinal Study of Parents and Children; UKB, UK Biobank; OR, odds ratio.

In multivariable models adjusted for age, sex, smoking, education and deprivation, per SD higher BMI, the OR for SARS-CoV-2(+) was 1.08 (95% CI: 0.96, 1.21) compared with SARS-CoV-2(−) and 1.10 (95% CI: 0.98, 1.23) compared with ‘everyone else’ (Figure 3). When stratifying by sex, results were similar in females but imprecisely estimated in males (Supplementary Figure 2a, available as Supplementary data at *IJE* online).

#### UKB

##### BMI and risk of SARS-CoV-2 infection

In multivariable models adjusted for age, sex, smoking status, educational attainment and deprivation index, per SD higher BMI, the OR for SARS-CoV-2(+) was 1.09 (95% CI: 1.03, 1.16) compared with SARS-CoV-2(−) and 1.26 (95% CI: 1.21, 1.32) compared with ‘everyone else’ (Figure 3). When stratifying by sex, results were similar between females and males (Supplementary Figure 2b, available as Supplementary data at *IJE* online).

##### BMI and risk of death-with-COVID-19

In multivariable models (adjusted as above), per SD higher BMI, the OR for death-with-COVID-19 was 1.16 (95% CI: 1.03, 1.31) compared with SARS-CoV-2(+) and 1.40 (95% CI: 1.27, 1.55) compared with ‘everyone else’ (Figure 3). When stratifying by sex, point estimates were slightly higher in females compared with males (Supplementary Figure 2b, available as Supplementary data at *IJE* online).

#### Simulation results

All simulations using the confounder adjusted model in the full sample (i.e. no selection) were unbiased (‘All participants, confounder adjusted’ in Tables 1 and 2), with coverage between 93.3% (MCSE=0.79) and 95.8% (MCSE=6.3).

**Table 1:** Results of simulations of SARS-CoV-2 infection based on the Avon Longitudinal Study of Parents and Children (ALSPAC)

			Bias (MCSE) or coverage (MCSE) of estimated effect of BMI on SARS-CoV-2 infection		
			SARS-CoV-2(+) vs SARS-CoV-2(−)		SARS-CoV-2(+) vs ‘everyone else’
Performance measure	Effect of BMI on SARS-CoV-2 infection	Interaction size of effect of BMI with SARS-CoV-2 infection on selection	All participants, confounder adjusted	Selected subsample, confounder adjusted	All participants
Bias	OR = 1	No interaction	−0.0017 (0.0010)	−0.0002 (0.0019)	0.0213 (0.0016)
		Plausible	0.0001 (0.0010)	0.0517 (0.0018)^$^	0.0595 (0.0015)
		Extreme	0.0001 (0.0010)	**0.1343** (0.0019)	**0.1194** (0.0015)
	OR = 3	No interaction	−0.0001 (0.0012)	0.0090 (0.0024)	−0.0802 (0.0017)
		Plausible	0.0018 (0.0012)	0.0652 (0.0024)	−0.0353 (0.0016)
		Extreme	0.0018 (0.0012)	**0.1479** (0.0025)	0.0283 (0.0016)
Coverage	OR = 1	No interaction	0.945 (0.0072)	0.944 (0.0073)	0.922 (0.0085)
		Plausible	0.958 (0.0063)	0.851 (0.0113)	**0.761** (0.0135)
		Extreme	0.958 (0.0063)	**0.369** (0.0153)	**0.297** (0.0144)
	OR = 3	No interaction	0.954 (0.0066)	0.948 (0.0070)	**0.667** (0.0149)
		Plausible	0.955 (0.0066)	0.886 (0.0101)	0.914 (0.0089)
		Extreme	0.955 (0.0066)	**0.543** (0.0158)	0.933 (0.0079)

Bias given is the difference in the estimated vs true effect (log odds ratio) of BMI on SARS-CoV-2 infection. Coverage is the proportion of simulation repetitions with confidence intervals containing the true effect. Sample sizes: all *N* = 14 849; selected subsample *n* ∼ 1450. Interaction magnitudes: no interaction: log risk ratio (RR)=0; plausible interaction: log RR = 0.135; extreme interaction: log RR = 0.135.

Example biases: A bias of 0.0517^$^ when no effect of BMI on SARS-CoV-2 infection (plausible scenario) is equivalent to an estimated odds ratio of 1.05 per 1-SD higher BMI (compared with true odds ratio = 1). Results shown in bold are those with concerning bias or coverage, defined as absolute bias >0.1, or coverage <0.8.

For reference, in the column ‘All participants, confounder adjusted’, we present results in which no bias would be expected based on a scenario simulated with no missing data and with regression models fully adjusted for confounders.

Histograms of simulation results are shown in Supplementary Figure 3 (available as Supplementary data at *IJE* online). Full results including unadjusted associations are given in Supplementary Table 6 (available as Supplementary data at *IJE* online).

BMI, body mass index; OR, odds ratio; MCSE, Monte Carlo standard error (across 1000 repetitions).

**Table 2:** Results of simulations of SARS-CoV-2 infection and death-with-COVID-19 based on UK Biobank data

(a) Results of simulations estimating effect of BMI on SARS-CoV-2 infection					
			Bias (MCSE) or coverage (MCSE) of estimated effect of BMI on SARS-CoV-2 infection		
			SARS-CoV-2(+) vs SARS-CoV-2(−)		SARS-CoV-2(+) vs ‘everyone else’
Performance measure	Effect of BMI on SARS-CoV-2 infection	Interaction size of effect of BMI with SARS-CoV-2 infection on selection	All participants, confounder adjusted	Selected subsample, confounder adjusted	All participants
Bias	OR = 1	No interaction	0.0001 (0.0003)	0.0020 (0.0013)	**0.1637** (0.0012)
		Plausible	0.0001 (0.0003)	−**0.1614** (0.0013)^$^	0.0259 (0.0012)
		Extreme	0.0001 (0.0003)	−**0.2440** (0.0014)	−0.0386 (0.0012)
	OR = 3	No interaction	0.0002 (0.0003)	0.0046 (0.0015)	0.0604 (0.0011)
		Plausible	0.0001 (0.0003)	−**0.1599** (0.0015)	−0.0702 (0.0011)
		Extreme	0.0001 (0.0003)	−**0.2427** (0.0015)	−**0.1305** (0.0011)
Coverage	OR = 1	No interaction	0.956 (0.0065)	0.961 (0.0061)	**0.012** (0.0034)
		Plausible	0.947 (0.0071)	**0.030** (0.0054)	0.901 (0.0094)
		Extreme	0.947 (0.0071)	**0.000** (0.0000)	0.834 (0.0118)
	OR = 3	No interaction	0.938 (0.0076)	0.950 (0.0069)	**0.608** (0.0154)
		Plausible	0.946 (0.0071)	**0.072** (0.0082)	**0.493** (0.0158)
		Extreme	0.946 (0.0071)	**0.002** (0.0014)	**0.046** (0.0066)

(b) Results of simulations estimating effect of BMI on death-with-COVID-19					
			Bias (MCSE) or coverage (MCSE) of estimated effect of BMI on death-with-COVID-19		
			Death-with-COVID-19 vs SARS-CoV-2(+) not resulting in death-with-COVID-19		Death-with-COVID-19 vs ‘everyone else’
Performance measure	Effect of BMI on death-with-COVID-19	Interaction size of effect of BMI with SARS-CoV-2 infection on selection	SARS-CoV-2(+) subsample, confounder adjusted	SARS-CoV-2(+) and assessed subsample, confounder adjusted	All participants
Bias	OR = 1	No interaction	−0.0013 (0.0017)	−**0.1652** (0.0022)	−0.0018 (0.0016)
		Plausible	−0.0004 (0.0016)	−0.0233 (0.0022)	−0.0004 (0.0015)
		Extreme	−0.0004 (0.0016)	0.0423 (0.0022)	−0.0004 (0.0015)
	OR = 3	No interaction	0.0012 (0.0019)	−**0.1525** (0.0027)	−**0.1404** (0.0016)
		Plausible	0.0016 (0.0018)	−0.0100 (0.0028)^#^	−**0.1381** (0.0015)
		Extreme	0.0016 (0.0018)	0.0564 (0.0028)	−**0.1381** (0.0015)
Coverage	OR = 1	No interaction	0.951 (0.0068)	**0.326** (0.0148)	0.945 (0.0072)
		Plausible	0.952 (0.0068)	0.935 (0.0078)	0.957 (0.0064)
		Extreme	0.952 (0.0068)	0.909 (0.0091)	0.957 (0.0064)
	OR = 3	No interaction	0.933 (0.0079)	**0.535** (0.0158)	**0.204** (0.0127)
		Plausible	0.953 (0.0067)	0.952 (0.0068)	**0.190** (0.0124)
		Extreme	0.953 (0.0067)	0.916 (0.0088)	**0.190** (0.0124)

Simulations based on UK Biobank polymerase chain reaction (PCR) test results from national testing. Bias given is the difference in the estimated vs true effect (log odds ratio) of BMI on (a) SARS-CoV-2 infection and (b) death-with-COVID-19. Coverage is the proportion of simulation repetitions with confidence intervals containing the true effect.

Sample sizes: (a) All *N* = 421 027; selected subsample *n* ∼ 18 000 and (b) SARS-CoV-2(+) subsample *N* ∼ 13 300K; SARS-CoV-2(+) and assessed subsample *n* ∼ 1600; whole sample *N* = 421 037. Interaction magnitudes: no interaction: log risk ratio (RR)=0; plausible interaction: log RR = 0.162; extreme interaction: log RR = 0.245.

For reference, in the column ‘All participants, confounder adjusted’, we present results in which no bias would be expected based on a scenario simulated with no missing data and with regression models fully adjusted for confounders. However, in the analyses of death-with-COVID-19 in Table 2(b), we may have bias in the reference scenario (column ‘SARS-CoV-2(+) subsample, confounder adjusted’) due to induced statistical interaction between death-with-COVID-19 and each other determinant of selection.

Example biases: a bias of −0.1614^$^ when no effect of BMI on SARS-CoV-2 infection (plausible scenario) is equivalent to an estimated odds ratio of 0.85 per 1-SD higher BMI (compared with true odds ratio = 1). A bias of 0.0564^#^ when BMI effect on death-with-COVID-19 is OR = 3 is equivalent to an estimated odds ratio of 3.17 (compared with true odds ratio=3). Results shown in bold are those with concerning bias or coverage, defined as absolute bias>0.1, or coverage<0.8.

Histograms of simulation results are shown in Supplementary Figure 5 (available as Supplementary data at *IJE* online). Full results including unadjusted associations are given in Supplementary Table 7 (available as Supplementary data at *IJE* online).

BMI, body mass index; OR, odds ratio; MCSE, Monte Carlo standard error (across 1000 repetitions).

### Simulations estimating the association of BMI with SARS-CoV-2 infection and death-with-COVID-19 outcomes

#### ALSPAC

The results of our simulations of SARS-CoV-2 infection based on ALSPAC are shown in Table 1 (histograms of estimates shown in Supplementary Figure 3, available as Supplementary data at *IJE* online).

##### Results for SARS-CoV-2(+) vs SARS-CoV-2(−) outcome (selected subsample)

As expected for analyses in the selected subsample, when assuming independent effects of BMI and SARS-CoV-2 infection on selection (i.e. no additive interaction on the log probability scale), estimates were unbiased when assuming no effect of BMI on SARS-CoV-2 infection (Supplementary Figure 4a, available as Supplementary data at *IJE* online, illustrates why this is the case for analyses using logistic regression). Unexpectedly, when assuming an effect of BMI on SARS-CoV-2 infection, we found positive bias. However, this bias disappeared when increasing the sample size, suggesting it was due to near separation (when a combination of covariates almost perfectly predicts the outcome)^23^^,^^24^ rather than bias due to use of a non-random subsample (see Supplementary Table 8, available as Supplementary data at *IJE* online). In the scenarios with an interaction effect of BMI and SARS-CoV-2 infection on being assessed, bias was positive and strengthened as the magnitude of the interaction increased.

##### Results for SARS-CoV-2(+) vs ‘everyone else’ outcome (whole sample)

When there was no interaction in the effects of BMI and SARS-CoV-2 infection on being assessed (i.e. in the presence of misclassification bias only), bias was positive when there was no effect of BMI on SARS-CoV-2 infection [expected ORs of exp(0.0213)=1.02 compared with a true odds ratio of 1] but negative when there was an effect {expected ORs=exp[ln(3)–0.0802]=2.77 compared with a true OR=3}. When there was an interaction in the effects of BMI and SARS-CoV-2 infection on being assessed, bias became less negative/more positive with increasing magnitude of the (positive) interaction (i.e. increasing amounts of selection bias in addition to misclassification bias). For illustration, assuming BMI does not affect SARS-CoV-2 infection, we estimated a mean bias of 1.06 for the plausible interaction effect size and 1.13 for extreme interaction effect size.

Coverage varied greatly depending on the scenario, ranging between 36.9% and 94.8% for the SARS-CoV-2(+) vs SARS-CoV-2(−) outcome, and between 29.7 and 93.3% for the SARS-CoV-2(+) vs everyone outcome.

#### UKB

The results of our simulations of SARS-CoV-2 infection based on UKB data are shown in Table 2a (histograms of estimates shown in Supplementary Figure 5a, available as Supplementary data at *IJE* online) and those of death-with-COVID-19 are shown in Table 2b (histograms of estimates shown in Supplementary Figure 5b, available as Supplementary data at *IJE* online).

##### Results for SARS-CoV-2(+) vs SARS-CoV-2(−) outcome definition (selected subsample)

Results for the ‘no interaction’ scenario were similar to those in ALSPAC (Supplementary Table 9, available as Supplementary data at *IJE* online, for results with larger sample). In the scenarios with an interaction effect of BMI and SARS-CoV-2 infection on being assessed, we found negative bias (opposite to ALSPAC because the interaction was in the opposite direction) that strengthened as the interaction magnitude increased.

##### Results for SARS-CoV-2(+) vs ‘everyone else’ outcome definition (whole sample)

Bias was positive when there was no interaction in the effect of BMI and SARS-CoV-2 infection on being tested, i.e. with misclassification bias only [e.g. expected ORs of exp(0.16)=1.17 compared with a true odds ratio of 1]. As the magnitude of the (negative) interaction effect increased (i.e. increasing amounts of selection bias in addition to misclassification bias), bias became less positive/more negative [e.g. expected ORs of exp(0.0259)=1.03 and exp(–0.0386)=0.96 for the plausible and extreme interaction effect sizes, compared with a true odds ratio of 1].

As with the ALSPAC simulations, coverage varied greatly depending on the scenario.

##### Results for death-with-COVID-19 vs SARS-CoV-2(+)

When assuming no interaction effect of BMI and SARS-CoV-2 infection on being tested (but with an interaction of death-with-COVID-19 with each other determinant of selection), estimates had negative bias and poor coverage [e.g. expected ORs of exp(–0.1652)=0.85 compared with a true odds ratio of 1 and 32.6% (MCSE=1.48) coverage when assuming no effect of BMI on death-with-COVID-19]. When including an interaction, bias became more positive and coverage improved [e.g. expected ORs of exp(–0.0233)=0.98 compared with a true odds ratio of 1 with 93.5% (MCSE=0.78) coverage for the plausible scenario with no effect of BMI on death-with-COVID-19].

##### Results for death-with-COVID-19 vs ‘everyone else’

We found little evidence of bias and good coverage when there was no effect of BMI on death-with-COVID-19 [e.g. bias=–0.0004 (MCSE=0.0015), coverage=95.7% (MCSE=0.64) for the ‘plausible’ interaction magnitude] and negative bias with poor coverage when BMI affected death-with-COVID-19 (OR=3) across the different interaction magnitudes [e.g. bias=–0.1381 (MCSE=0.0015), coverage=19.0% (MCSE=1.24) for the ‘plausible’ interaction magnitude].

In general, the comparison group with the least bias (and hence better coverage) depended on the particular assumptions used for the data-generating mechanism. For example, when assuming no effect of BMI on SARS-CoV-2 infection and plausible interaction magnitude (for the effect of BMI and infection on selection), estimates of bias were comparable in the scenario based on ALSPAC data [bias=0.0517 (MCSE=0.0018) comparing SARS-CoV-2(+) to SARS-CoV-2(−) vs 0.0595 (MCSE = 0.0015) comparing SARS-CoV-2(+) to ‘everyone else’]. In contrast, in the scenario based on UKB, the SARS-CoV-2(+) vs SARS-CoV-2(−) (selected subsample only) outcome definition had greater bias compared with the SARS-CoV-2(+) vs ‘everyone else’ definition [bias=–0.1614 (MCSE = 0.0013) vs 0.0259 (MCSE = 0.0012)].

Bias-eliminated coverage were all near to 0.95 [bias-eliminated coverage for all confounder adjusted estimates between 93.6% (MCSE = 0.77) and 96.3% (MCSE = 0.6)], confirming that the poor coverage in some scenarios was driven solely by bias (Supplementary Tables 6 and 7, available as Supplementary data at *IJE* online).

## Discussion

In this study, we investigated the potential impact of selection on the association between BMI and COVID-19 outcomes using empirical analyses and simulations. In both ALSPAC and UKB, a broad range of characteristics were related to selection, sometimes in opposite directions (e.g. more-educated participants were more likely to be assessed for SARS-CoV-2 infection in ALSPAC but less likely in UKB). In empirical analyses, estimates were imprecise in ALSPAC but UKB analyses suggested that higher BMI was associated with higher odds of SARS-CoV-2 infection and death-with-COVID-19, and the magnitude tended to be sensitive to the choice of comparison group. In simulations, the magnitude and direction of bias estimated varied widely depending on the specific data-generating mechanism (e.g. the magnitude of the interacting effect on selection) and the comparison group used.

The simulation results can be used to assess the empirical results in the context of potential biases. For instance, in UKB we estimated a larger positive association of BMI on SARS-CoV-2 infection using the SARS-CoV-2(+) vs ‘everyone else’ definition compared with SARS-CoV-2(+) vs SARS-CoV-2(−). We can compare this with the simulation results when assuming an interacting effect of BMI and infection on selection (which we believe to be more plausible than assuming no effect). Of these, results assuming no effect of BMI on SARS-CoV-2 infection are inconsistent with the empirical results as they showed negative bias, which would result in OR<1. In our simulation scenarios assuming a positive effect of BMI on SARS-CoV-2 infection (again assuming an interacting effect of BMI and infection on selection) we found negative bias that was smaller for the ‘everyone else’ compared with the SARS-CoV-2(−) comparison group. This provides further support for a positive association between BMI and SARS-CoV-2 infection, as a negative bias would mean the true effect is greater than the empirical estimates (i.e. further from the null).

In general, the bias for the SARS-CoV-2 infection simulations for both comparison groups [i.e. SARS-CoV-2(−) or ‘everyone else’] depended on the direction and magnitude of the interaction effect of BMI and infection on selection. For the ‘everyone else’ comparison group, differential selection-induced misclassification, where the SARS-CoV-2(+) non-assessed participants were included in the comparison group, means that the direction of the bias overall also depends on the BMI and infection distributions and the effects of selection across these. Furthermore, for the death-with-COVID-19 analyses, including all participants who died with COVID-19 statistically induces an interaction between death-with-COVID-19 and all other determinants of selection (e.g. BMI affects selection only in those who did not die with COVID-19 such the BMI’s effect on selection is modified by death-with-COVID-19), which also induces bias in the estimated effect of BMI on death-with-COVID-19. We provide further details and intuition for these biases in Supplementary Section 6 and Supplementary Figure 4 (available as Supplementary data at *IJE* online).

One of the strengths of this study lies in the use of two cohorts with contrasting sources of COVID-19 data (from questionnaires in ALSPAC and national registries in UKB). In addition, we used simulation parameters based on either cohort data or other secondary sources to try to reflect realistic scenarios. Key limitations include the fact that we were not able to estimate all simulation parameters with certainty and we focused solely on bias due to selective assessment of SARS-CoV-2 and COVID-19 status. Although the latter was motivated to illustrate the implications of selection bias due to such selective assessment, multiple selection mechanisms are likely to be simultaneously in place in studies exploring causes and consequences of SARS-CoV-2 and COVID-19 disease, which might bias results. As an example, ALSPAC has substantial loss to follow-up (41% were sent a questionnaire, of whom 57% returned it)^25^ and UKB has a low recruitment rate (5.5%).

We have defined our target populations as young (ALSPAC) and middle-aged and elderly (UKB) adults living in England in March 2020. For this to be a valid target population, we assume that young adults living in Avon and middle-aged and elderly adults recruited into UKB are generalizable to the UK in March 2020. The presence and/or magnitude of selection bias inherently relies on the definition of the target population. Different research questions using the same study data may define a different target population or may be subject to different selection pressures, which will be subject to different biases due to sample selection. Supplementary Section 7 (available as Supplementary data at *IJE* online) explains further details and limitations.

Given the logistic and ethical issues involving clinical trials, most evidence on risk and prognostic factors for the disease comes from observational studies^26^ but teasing apart causal from non-causal relationships in such studies is notoriously difficult due to confounding, selection and measurement error. Previous studies have identified several factors predicting selection for COVID-19 analytical subsamples.^25^^,^^27^^,^^28^ In agreement with our findings, studies have reported that higher BMI is associated with higher odds of SARS-CoV-2 infection and COVID-19 prognosis.^29–32^ However, our study indicates that the estimates reported in these studies may be impacted by selection bias. Our findings suggest that sample selection pressures can substantially differ between and within studies and may depend on a number of factors, such as the data-collection mechanism, sample ascertainment and characteristics of the target population. In addition, our results illustrate that bias due to sample selection and selection-induced misclassification can distort relationships between risk/prognostic factors and disease in an unpredictable way. This indicates that there is no ‘one-size-fits-all’ solution and individual studies should investigate whether and how selection pressures may bias their results, and consider sensitivity analyses that mitigate these biases (e.g. inverse probability weighting, multiple imputation).

**Figure 4:**
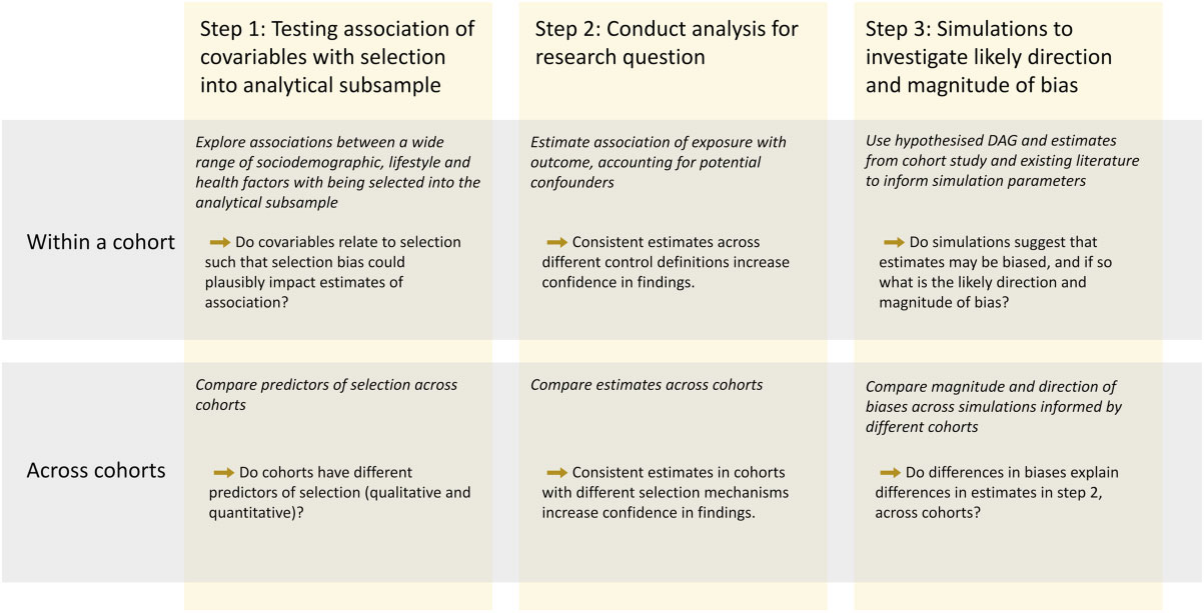
A general framework for investigating the impact of selection bias. Prior to Step 1, researchers should have developed a directed acyclic graph (DAG) for their defined research question. After Step 3, researchers may choose to use approaches that account for missing data, e.g. inverse probability weighting or imputation. Researchers may also want to integrate our framework with the Treatment And Reporting of Missing Data in Observational Studies framework (specifically Step 2: Examine the data).^33^

While in this study we have focused on assessing biases due to selection in studies of COVID-19, we have presented a general framework that is applicable to other research conducted on a much smaller subsample of the original cohort. This framework, illustrated in Figure 4, involves three components that each help to build a picture of the possible direction and magnitude of bias for a specific research population, cohort and subsample, and determine a level of confidence in results. We have provided substantial Supplementary information describing how researchers can design simulations to induce selection bias (Supplementary Sections 1–3, available as Supplementary data at *IJE* online). We provide code that can be adapted by other researchers wanting to apply our framework to their own research question.

## Ethics approval

Informed consent for the use of ALSPAC data collected via questionnaires and clinics was obtained from participants following the recommendations of the ALSPAC Ethics and Law Committee at the time (details and reference numbers of all ethics approvals can be found at http://www.bristol.ac.uk/media-library/sites/alspac/documents/governance/Research%20Ethics%20Committee%20approval%20references.pdf). The work we present here was approved by the ALSPAC Ethics and Law Committee under project B3543. UKB received ethical approval from the UK National Health Service’s National Research Ethics Service (ref. 11/NW/0382). This research was conducted under UKB application number 16729.

## Supplementary Material

dyac221_Supplementary_Data

## Data Availability

Bona fide researchers can apply to ALSPAC and UKB to use these data.
